# Control of Separation and Diameter of Ag Nanorods through Self-organized Seeds

**DOI:** 10.1038/srep16826

**Published:** 2015-11-20

**Authors:** Paul R. Elliott, Stephen P. Stagon, Hanchen Huang

**Affiliations:** 1 Mechanical Engineering, University of Connecticut, Storrs, CT 06269, USA; 2 Mechanical Engineering, University of North Florida, Jacksonville, FL 32224, USA; 3 Mechanical and Industrial Engineering, Northeastern University, Boston, MA 02115, USA

## Abstract

This paper proposes a mechanism of controlling the diameter and separation of metallic nanorods from physical vapor deposition through self-organized seeds and experimentally demonstrates the feasibility using Ag as the prototype metal, In as the seed, and Si the substrate. Being non-wetting on Si substrates, deposited In atoms self-organize into islands. Subsequently deposited Ag atoms attach to In islands, rather than to Si substrates, due to preferential bonding and geometrical shadowing. The experimental results show that self-organized In seeds of 5 nm nominal thickness give rise to the best separation and the smallest diameter of Ag nanorods.

Small and well-separated metallic nanorods have unique properties that are advantageous for various applications, such as surface-enhanced Raman scattering (SERS)^1^,^2^ and metallic glue^3^. For SERS applications, structures with close proximity yet good separation produce the highest signal enhancement^4^. For metallic glue, densely packed metallic nanorods require about 300 °C to successfully form bonds^5^. Alternatively, when the nanorods have adequate separation the interpenetration of nanorods on opposing substrates is possible. Once in contact, fast diffusion on the metallic nanorod surfaces drives the interpenetrated nanorods to coarsen into a continuous film, or metallic glue. It is through small and well-separated metallic nanorods that the first room temperature and low-pressure metallic glue technologies have been enabled^3^.

Recognizing the advantages of small diameter and sufficiently large separation for metallic nanorods, one naturally asks how to achieve them in a controllable manner. As discussed in our earlier review^6^, physical vapor deposition (PVD) allows for the growth of metallic nanorods of high purity and by simple processes. Therefore, here we will focus on PVD processes in designing mechanisms to control the diameter and the separation of metallic nanorods. Recently developed theories on both the diameter^7^ and the separation^8^ of metallic nanorods serve as the starting point of this design.

According to the theories^7^,^8^, the diameter and the separation of nanorods both, in a coupled fashion, decrease with the increase of deposition rate and/or the decrease of substrate temperature, although following different scaling laws. As a result, it is desirable to decouple the variation of the diameter with that of the separation. For this purpose, we propose a new mechanism to decouple the variations by introducing self-organized seeds before the growth of the nanorods. The separation of nanorods derives from the separation of the seeds and therefore depends on the diffusion kinetics of the seed atoms, instead of that of the nanorod atoms. In contrast, the diameter of the nanorods still depends on the diffusion kinetics of the nanorod atoms. In the following, we present the proposal by using silver (Ag) as the prototype nanorod metal, indium (In) as the self-organized seeds, and silicon (Si) with a native oxide layer as the substrate. This choice of materials takes advantage of two pieces of knowledge: (1) In is non-wetting on Si substrates with native oxide^9^, and (2) Ag bonds more strongly to In than to the native oxide layer on Si^10^,^11^.

The proposed mechanism of control assumes PVD deposition with no interruption of vacuum, as shown in Fig. 1. At the initial stage, In is deposited onto a Si substrate and forms islands instead of a continuous film due to the non-wetting interaction; Fig. 1(a). Subsequently, Ag is deposited and Ag atoms preferentially bind to the In islands; Fig. 1(b). This occurs for two reasons. First, thermodynamically, Ag atoms bond more strongly to In than to the native oxide layer of Si^10^,^11^. Second, Ag atoms are more likely to land on the taller In islands than the Si substrate due to geometrical shadowing under the glancing angle deposition (GLAD) condition for nanorod growth^12^. As a result, the taller In islands serve as seeds to define the separation of Ag nanorods, and to affect the diameter of Ag nanorods; Fig. 1(c). To achieve large separation of the taller In islands, it is desirable to deposit more In atoms so only fewer In islands survive in the tallest group. The large separation is a result of shadowing from adjacent seeds during the early stages of deposition. The seeds that are strongly shadowed receive little or no flux early on and do not grow tall. To achieve small diameter Ag nanorods, it is desirable to deposit fewer In atoms so that the diameters of In islands are sufficiently small to facilitate the Mode II growth according to the theory^13^. As a tradeoff, there is an optimal amount of In deposition.

## Results

In this Report, we use PVD to demonstrate that the proposed mechanism of control is feasible. Further, through scanning electron microscopy (SEM) and transmission electron microscopy (TEM) characterization, we show that self-organized In seeds of 5 nm in nominal thickness, which is the thickness if a dense film were to form on a normally oriented substrate, leads to optimal diameter and separation of Ag nanorods.

**Figure 2:**
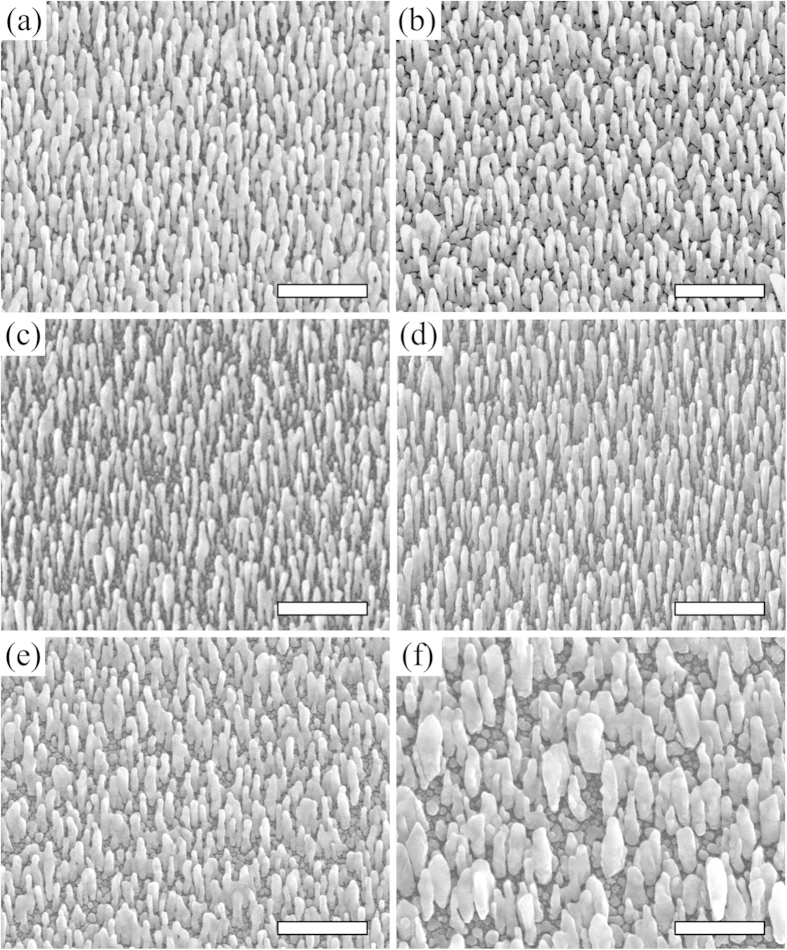
Ag nanorods on In seeds. SEM images, taken normal to the substrate, of Ag nanorods produced by glancing angle PVD on In seeds of various sizes. Indium deposition amounts are (**a**) 0 nm, (**b**) 1 nm, (**c**) 5 nm, (**d**) 10 nm, (**e**) 50 nm, and (**f**) 100 nm; in nominal thickness. The scale bars are 500 nm.

Figure 2 shows Ag nanorods when grown with varying amounts of In seed atoms, holding all other variables constant. As proposed, indeed the control of both the separation and the diameter of nanorods is possible through seeding. Further, the In seeds of 5 nm in nominal thickness give rise to the largest separation between adjacent nanorods and the smallest diameter of individual Ag nanorods; Fig. 2(c). When even larger amounts of In, such as 10 nm in nominal thickness, are used as seeds the Ag nanorods have even larger separation, but the diameter also increases.

**Figure 3:**
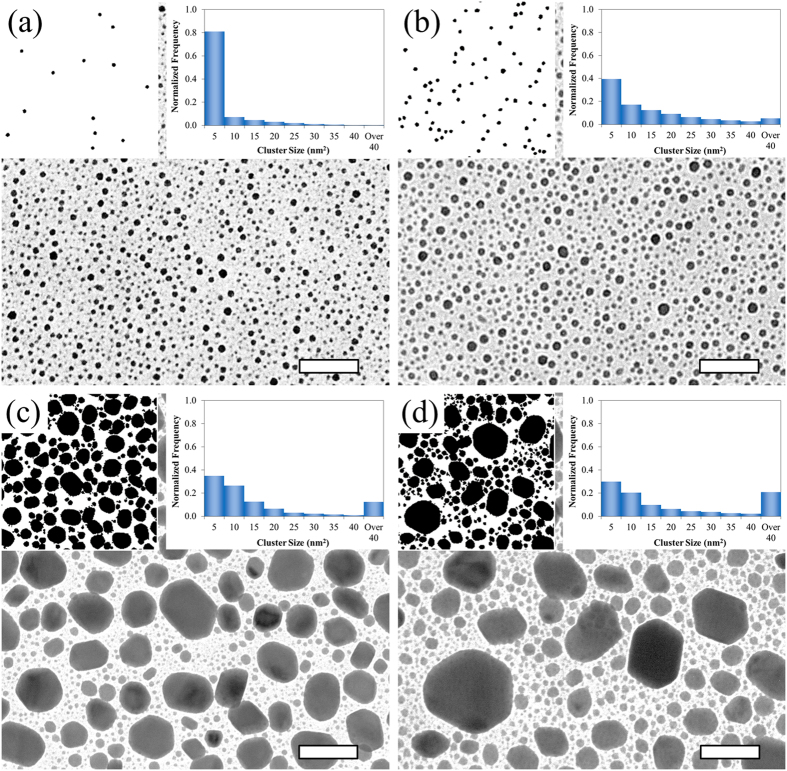
In seeds of various sizes. TEM images of In seeds of (**a**) 5 nm, (**b**) 10 nm, (**c**) 50 nm, and (**d**) 100 nm on a silicon dioxide substrate; in nominal thickness. Left insets show processed images of In seeds with diameter being 10 nm or larger. Right insets show a histogram of the size distribution of seeds. The scale bars are 50 nm.

**Figure 4:**
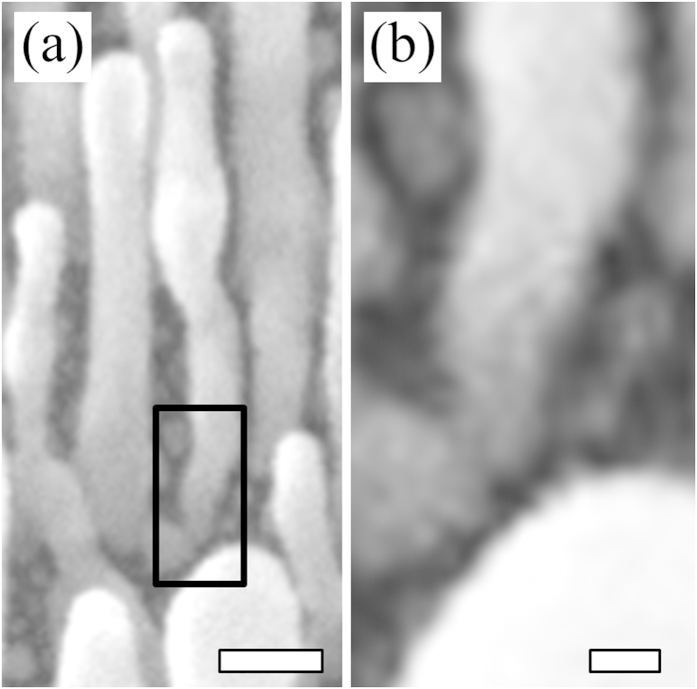
Close view of nanorod diameter growth from seed. (**a**) Expanded view of Fig. 2(c), showing the initial diameter of Ag nanorod being ~7 nm as highlighted in the black box. Scale bar is 50 nm. (**b**) View of boxed area at higher magnification. Scale bar is 10 nm.

Going one step further, we experimentally analyze the size distribution of the In seeds. The densities of nanorods, from Fig. 2, inform us of the density of relevant In seeds on which Ag nanorods successfully develop. From Fig. 2(c), the density of nanorods, or that of relevant In seeds, is ~52/μm^2^. The relevant In seeds must be the largest ones. To reach the density of 52/μm^2^ for the case of In deposition of 5 nm in nominal thickness, the diameter of such seeds must be greater than or equal to 7 nm; as shown in Fig. 3(a). This value was determined by matching the number of nanorods in a given area to the number of seeds larger than the critical size for the same area. The left insets of Fig. 3 show the In seeds of 7 nm in lateral dimension or larger. When an even smaller amount of In is deposited, the In seeds are too small to be effective, as Fig. 2(a,b) indicate. As the deposition of In reaches 10 nm in nominal thickness, the relevant In seeds are close together. As a result, the Ag nanorods that develop on the In seeds form bridges; Fig. 2(d). Further increase of In deposition leads to even larger In seeds, which are fewer and thereby more separated; Fig. 3(c,d). This is detailed by the right insets in Fig. 3 which show a histogram of the size distribution of seeds. The increased separation is accompanied by an increased diameter of nanorods; Fig. 2(e,f). To strengthen the analyses of size distributions of In seeds, we note that the smallest diameter of relevant In seeds in Fig. 3(a), 7 nm, corresponds to the initial diameter of Ag nanorods; as shown in Fig. 4.

**Figure 5:**
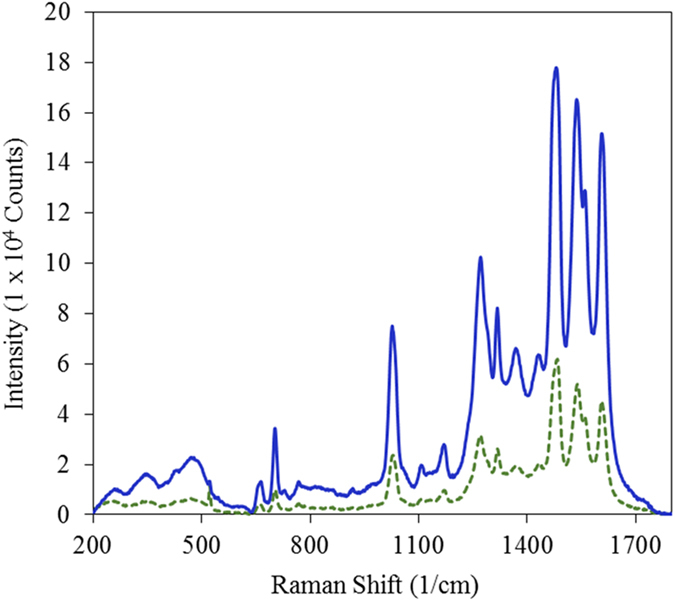
Surface-enhanced Raman spectroscopy. Spectra taken of N719 dye on Ag nanorods with no seed layer (green dotted) and with In 5 nm seed layer (solid blue).

To demonstrate the significance of the change in morphology, SERS tests were performed. To test the speculation of performance improvement we compare Ag nanorods grown without seeds to those grown with 5 nm In seeds, which are chosen as they have the smallest diameter and best separation. Figure 5 shows the SERS spectra obtained from sensitizing Ag nanorod substrates in low concentrations of N719 dye. With background deleted, the Ag nanorods grown with 5 nm of In seeds demonstrate a three fold enhancement of the Raman signal over the Ag nanorods grown without seeds.

**Figure 6:**
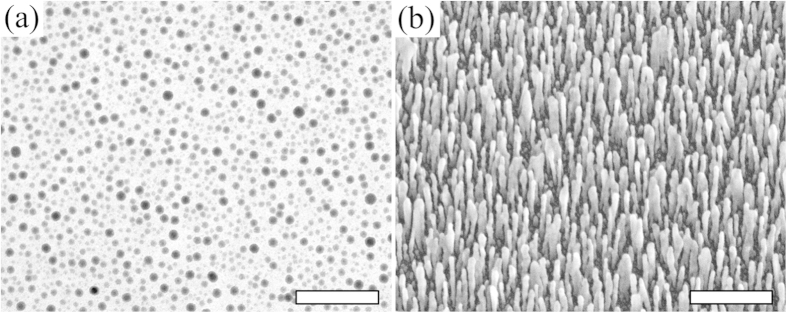
Seeds of Sn and Ag nanorods grown on same. (**a**) TEM image of Sn seeds of 10 nm nominal thickness. The scale bar is 100 nm. (**b**) SEM image, taken normal to substrate, of Ag nanorods from glancing angle PVD on Sn seeds; all other conditions are the same as in Fig. 2. The scale bar is 500 nm.

To further test the generic nature of our proposed mechanism we replace In by tin (Sn). Post transition metals and metalloids are likely to provide the best cluster forming effects, with examples including tin, antimony, and lead, which have been used as surfactants in metal film growth^14^. Being surfactant, these transition metals and metalloids will not easily mix with Ag, so as to keep the nanorods relatively pure Ag. In addition, these surfactants are also non-wetting on the native oxide layer of a Si substrate and bond strongly with Ag^10^,^11^. Like In, Sn is indeed non-wetting on the Si substrate as shown in Fig. 6(a). Figure 6(b) shows Ag nanorods on the Sn seeds, which are smaller and better separated than those in Fig. 2(a) without seeds. That is, our proposed mechanism is generic in nature, as evidenced by the similarities of seeding effects with In and Sn. While the method is generic, the diffusion and islanding of Sn on the substrate differs from In. As a result, the seeding of 10 nm Sn, in contrast to 5 nm In, produces the smallest diameter and best separation.

## Discussion

In passing, we compare the proposed mechanism of using self-organized seeds to control separation and diameter with those in the literature. At least three other mechanisms allow the introduction of seeds. They include lithography^15^, focused-ion-beam (FIB) patterning^16^, and pre-patterning with polymer clusters^17^. Our proposal offers the advantage of simplicity when compared to lithography, not requiring chemical baths or a clean room environment. It is also low cost and rapid for large areas when compared to FIB milling. Unlike pre-patterning with polymer clusters, our method occurs entirely in the PVD vacuum chamber and can be performed just prior to the deposition for the growth of the nanorods, without a vacuum break, in minutes.

In summary, we have proposed a mechanism of using self-organized seeds to control the diameter and the separation of metallic nanorods in a decoupled fashion during PVD. Further, using Ag as the prototype metal, In as the seed, and Si as the substrate we have experimentally demonstrated the feasibility of this proposal. The In seeds of 5 nm in nominal thickness give rise to the largest separation and the smallest diameter of Ag nanorods. In addition, the substitution of In with Sn indicates that the proposed mechanism is generic.

## Methods

The deposition process takes place in a vacuum chamber using electron beam PVD. The source materials, in two separate Fabmate^®^ crucibles, are 99.99% In and 99.99% Ag (Kurt J. Lesker). A Si {100} wafer (Nova Electronic Materials) with native oxide layer is ultrasonically cleaned in acetone, ethanol, and de-ionized water before being placed onto a holder on the top of the vacuum chamber. The holder is set at 85 degrees with respect to the source normal for both the deposition of In and Ag. The vacuum chamber is approximately 40 cm tall and 25 cm in diameter with the source to substrate distance being approximately 35 cm. The base pressure of the chamber is 1 × 10^−5^ Pa, and the working pressure is 1 × 10^−3^ Pa. The rate of deposition is monitored using a quartz crystal microbalance, which is normal to the flux and adjacent to the substrate. The power of the electron beam is controlled to maintain a deposition rate of 0.05 nm/s for In and 1.0 nm/s for Ag.

The characterization takes place *ex situ*. Immediately after the growth is complete the substrates are removed from the deposition chamber and characterized using a Quanta 250 Field Emission SEM (FEI). To examine the In seeds, TEM grids with silicon dioxide support films of 8 nm in thickness are placed into the chamber as the substrate (Ted Pella). In is deposited onto the grids and the In seeds are then characterized using a Tecnai T12 TEM (FEI). SERS data were taken with a Renishaw Raman 2000 using a 514 nm laser operating with a 50% power reducing filter with 10 s acquisition averaged over 3 runs at 50× magnification. Samples were soaked in 5 mM N719 Ruthenium dye (Sigma) in methanol (Sigma) for 12 hours then rinsed with methanol.

## Additional Information

**How to cite this article**: Elliott, P. R. *et al.* Control of Separation and Diameter of Ag Nanorods through Self-organized Seeds. *Sci. Rep.* **5**, 16826; doi: 10.1038/srep16826 (2015).

## Figures and Tables

**Figure 1:**
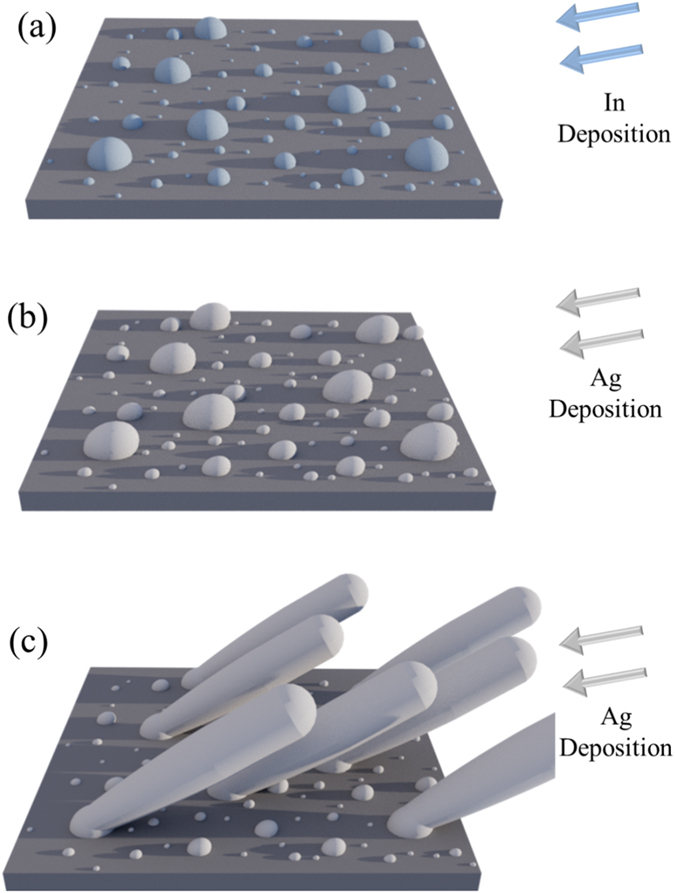
Nanorod development from seeds. A series of schematics showing Ag nanorod growth on In seeds deposited onto a Si substrate. (**a**) In (blue) is deposited at a high glancing angle onto a Si substrate (dark gray) forming In islands as seeds of various sizes. (**b**) Ag (light gray) is subsequently deposited at a high glancing angle, causing larger seeds to grow more. (**c**) Ag nanorods grow with their separation being that of the larger In seeds.
